# Association between psychological distress and mental help-seeking intentions in international students of national university of Singapore: a mediation analysis of mental health literacy

**DOI:** 10.1186/s12889-023-17346-4

**Published:** 2023-11-28

**Authors:** Fanmin Zeng, Wong Chee Meng John, Dan Qiao, Xueli Sun

**Affiliations:** 1 Mental Health Education Centre of Southwest Minzu University, Chengdu, China; 2 Department of Psychological Medicine, National University Hospital & National University of Singapore, Singapore, Singapore; 3 International Office, Wuhan University of Technology, Wuhan, Hubei China; 4 Mental Health Centre of West China Hospital in Sichuan University, Chengdu, China

**Keywords:** Mental help-seeking intentions, Mental health literacy, Psychological distress, International students

## Abstract

**Background:**

International students encounter a wide range of challenges that can have a significant impact on their mental health. Seeking help is one of the primary means of managing mental health, and more attention is required. This study aimed to investigate the psychological distress(PD), mental health literacy(MHL), and mental help-seeking intentions(MHSI) in international students of National University of Singapore(NUS), to explore the correlation between the three and to verify the mediating role of MHL in PD and MHSI.

**Methods:**

This cross-sectional study was conducted between May and July 2023 using the Mental Help Seeking Intention Scale(MHSIS), Patient Health Questionnaire-9(PHQ-9), Generalized Anxiety Disorder-7**(**GAD-7), and Mental Health Literacy Scale(MHLS). 281 international students(177 males, 104 females;) in NUS completed self-report questionnaires. SPSS 25.0 was applied to the data for descriptive analysis, Pearson correlation analysis and stepwise regression analysis. Mediation analysis fully for all potential confounding factors were conducted.

**Results:**

Significant correlations were found between PD, MHL and MHSI. MHLS- knowledge of how to seek professional information(MHLS- H) completely mediated the association of anxiety with MHSI[B=-0.271; 95% confidence interval(CI): (-0.067, 0.0037)]; MHLS- attitude that promote recognition or appropriate help-seeking behavior(stigma)(MHLS- H) partially mediated the association of PD with MHSI[B = 0.104, 95% CI: (0.008, 0.179)]; with mediating effects accounting for 100% and 24.847% of the total effect. In addition, demographic variables such as gender, years in Singapore and residence type mediated both the direct and indirect effect of the mediation model.

**Conclusions:**

MHL mediated both the direct and indirect effects on the association between PD and MHSI, especially, the mediator of the MHLS- H and MHL-A. It means that MHSI in this population can be improved by increasing MHL and thus the PD. The findings suggest that, such as providing information about how to access to professional services and promoting disorder recognition to the international students, may help them develop their psychological well-being and good mental health care decisions.

## Highlights

Prevalence of depression and anxiety symptoms was observed in 19.57% and 20.28% of the participants, respectively.

The MHSI displayed significant variations across demographic characteristics.

Depression, MHLS- A, MHLS- H, and gender were identified as significant predictors of MHSI.

A significant mediating effect of MHL was observed in the relationship between PD and MHSI.

## Introduction

Mental health among international students has garnered increasingly public concern recently. Numerous studies have documented the heightened risk of mental health challenges, such as loneliness, depression, and academic stress, faced by international students [1, 2]. These challenges may be compounded by language barriers, cultural disparities, and feelings of social isolation [3]. Compared to domestic students, international students are more susceptible to experience higher levels of depression and anxiety [4–6]. Paradoxically, despite the elevated prevalence of mental health issues within this population, there is a lower frequency of seeking professional psychological services [7, 8]. The researchers have explored the underlying reasons for this low professional service-seeking behavior among this group, identifying factors such as cultural stigma surrounding mental illnesses and their treatment, unfamiliarity with available services, and differences in help-seeking behavior [9, 10]. Given the potential impact of mental health on academic performance and overall well-being, it is crucial to assess and promote the mental health of international students to address these challenges [11].

The Mental Health Literacy(MHL) of an individual with mental health problems is crucial factor that could predict their attitudes toward seeking help [12]. MHL was first defined as “knowledge and beliefs about mental disorders which aid their recognition, management or prevention [13]” and consisted of six domains: “ability to recognize specific disorders or different types of psychological distress”, “knowledge and beliefs about risk factors and causes”; “knowledge and beliefs about self-help intervention” “knowledge and beliefs about professional help available” “attitudes which facilitate recognition and appropriate help-seeking(stigma)” and “knowledge of how to seek mental health information” [14]. MHL is a determinant of mental health that can facilitate the early detection of psychological problems and promote timely access to care [15]. Study has underlined the need to promote MHL throughout the life span and in different contexts, because it increases the quality of life of people [16]. The inadequacy of an individuals MHL may lead to an unwillingness to seek help, while the formers improvement has been shown to increase help-seeking intentions [17]. Previous research has shown that international students had a lower MHL score because of a different conceptualization of MHL relating to cultural diversity [18]. In addition, a nationwide MHL study in Singapore found a preference among the lay public to recommend informal sources of help such as friends and family for people [19] with mental illness [20].

Based on the theory of planned behavior(TPB), ones intention to perform a particular behavior is the central element, and it is also an intermediary in that attitude indirectly affects behavior [21]. Moreover, the intention is the direct premise of actual behavior [22]. Thus, improving mental help-seeking intention can promote further help-seeking behavior according to the TPB, and it is consistent with the findings of prior study [23]. Studying the intention to seek help for PD will, therefore, help to know participants intended plan for help that would have a vital role to access participants with PD. Recognizing this importance, researchers have extensively studied the relationship between help-seeking attitudes and related constructs. Of note, Mental Help-seeking intentions(MHSI) is an adaptive coping process that is an attempt to get external help to deal with a mental health concern some studies revealed that the university students intention to help for depressive symptoms was low which is, 1.32—3.72% [24]. However, another studies showed that the students intention to seek psychologist professional help for depression symptom was high, which is ranging from 1.7—7.3% [25]. International students reports more negative attitudes and lower intentions for seeking help for mental health concerns compared with domestic students [26]. Many international students believe that seeking psychological help is a sign of weakness and failure. Due to this self-stigmatization, they are more fearful of disclosing their problems and concerns to professional psychologists and re less willing to seek mental health services [27]. Research indicated that nonnative English speakers can find it difficult to understand mental health-related issues or express their concerns in a second language owing to fears of not being understood or feeling of embarrassment or shame [28]. As such, many Chinese-speaking international students preferred to handle the problem alone or rely on an intimate partner, family, or friends for help [29].

**Fig. 1:**
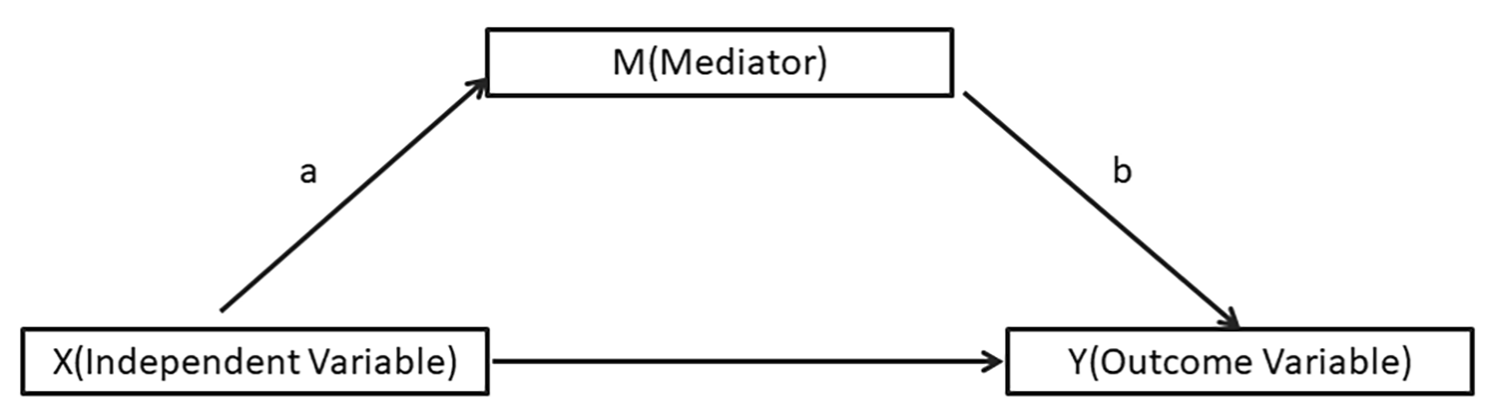
shows multi-path diagram for the relationship between an independent variable (X), an outcome variable (Y), and a mediator (M). In this diagram, there are three paths. Path **(a)** from the independent variable (X) to the mediator (M); path **(b)** form mediator (M) to the outcome variable (Y), and path (c) from the independent variable (X) to the outcome variable (Y). Path **(c)** represents the direct impact of (X) on (Y), while path **(a)**-**(b)** represents the indirect impact of (X) on (Y) mediated by (M) [30]

In summary, seeking help for mental health issues is the first step toward assessing the mental state, getting the proper diagnosis and subsequently undergoing the intervention and management of mental health by professionals. Despite the evidenced association between MHL and MHSI, we currently lack clarity on how different dimensions of MHL may interact with and influence MHSI in the internal students in NUS. However, the factor influencing MHSI need to be explored further, especially in the group of international students. Therefore, the aim of this study was to fist test whether MHL would mediate the relationship between PD and mental MHSI (Figs. 1). Then, the study would explore how the mediator of MHL on the relationship between the two variables, including which one is a stronger mediator of those mediating model. In addition, the study also sought to test the mediating role of the demographic factors, including gender, age, marital status, years in Singapore, degree level, residence type, economic status and the past mental illness history. This knowledge might help the universities to choose and implementing effective methods and programme which would ensure better mental health of the international students. Our hypothesis 1 is that MHL would mediate the relationship between PD and MHSI. The hypothesis 2 is that the demographic variables would medicate effect on the link between the PD and MHLI.

## Methods

### Ethical approval and informed consent

This study was approved by the National University of Singapore Institutional Review Board (NUS-IRB-114). Informed consent was obtained from all the subjects involved in the study.

### Study design and subjects

This cross-sectional study was conducted with international students in NUS between May to July 2023. The convenience sample included participants from NUS (age 18 to 60 years old). The inclusion criteria were that they were above 18 years old and who are foreigners enrolled in a part-time, full-time, exchange, or scholarship programme (including undergraduate, masters, doctoral and post-doctoral programme) in NUS. The exclusion criteria were international students in NUS who refused to participate in the study and less than 18 years old. We presented the questionnaire online, which was openly accessible to the international students at NUS. During the planned collect data period the 281 students who provided informed consent completed a questionnaire to measure their MHL (MHLS), psychological distress (PHQ-9,GAD-7), and MHSI (MHSIS). Before requesting that they complete the questionnaire, we informed participants of the purpose of the study, their right to refuse participation, and their right to withdraw from participation at any stage.

### Data sources and measurement

#### Demographic data

The Qualtrics Survey platform was used for administering the survey. The online survey consisted of demographic questions involving age, gender, marital status, length of stay in Singapore, degree level, residence type and economic status. A single Yes/No question of psychiatric diagnosis /treatment history was included to identify the horizontal continuum of mental health status and mental illness.

#### Mental help seeking intention scale (MHSIS)

This scale is an adapted version from Ajzens Theory of Planned Behaviour (2006) and was standardized by Dr Joseph H Hammer and Douglas Spiker in 2018 [31]. It is a 3-item instrument designed to measure respondents intention to seek help from a mental health professional if they had a mental health concern with responses ranging from strongly agree (7) to strongly disagree (1). A higher score indicates a greater intention to seek help. The finding supports the MHSIS measurement tool as a unidimentional instrument that produces a single total score [32]. Internal consistency (α’s > 0.87) and convergent evidence of validy for the MHSIS score has been documented in the form of significant positive associations between intention and both attitudes and subjective norms around seeking professional psychological help [33].

#### Patient health questionnaire-9 (PHQ-9)

The PHQ-9 was used as a self-administered, screening tool for assessment of the severity of depressive symptoms. PHQ-9 includes 9 items which assesses how often the subjects had been disturbed by any of the 9 items during the immediately preceding 2 weeks. Each item of PHQ-9 was scored on a scale of 0–3 (0 = not at all; 1 = several days; 2 = more than a week; 3 = nearly every day). The PHQ-9 total score ranges from 0 to 27 (score of 5–9 are classified as mild depression; 10–14 as moderate depression; 15–19 as moderately severe depression; ≥ 20 as severe depression) [34].The coefficient α was 0.78 [35].

#### Generalized anxiety disorder-7 (GAD-7)

The GAD-7 is a brief self-administered rating scale that assesses the severity of anxiety symptoms in the past 2 weeks [36]. The scale consists of 7 items that are statements about worry or somatic symptoms and are rated on a four-point Likert scale ranging from 1 (not at all) to 3 (nearly every day), for a total score of 0 to 21. A higher score indicates that anxiety symptoms are more severe (score of 5–9 are classified as mild anxiety; 10–14 as moderate anxiety; ≥ 15 as severe anxiety). The GAD-7 has been validated as a reliable measurement with a Cronbachs α coefficient of 0.92 [37].

#### Mental health literacy scale (MHLS)

It introduced by O Connor and Casey in 2015, assesses 3 fo 4 dimensions of MHL, including knowledge about mental disorders, stigma, and help-seeking behavior [38]. It is reported as both a valid and reliable tool with Cronbachs α coefficient of 0.873 and test-retest reliability (*r* = 0.797, p < 0.001) [38]. It has 35 questions with a 4 or 5 Likert scale. The scale includes six sub-dimensions: F1-Ability to recognize disease (item1-8), F2-Knowledge about how to seek information data (items 16–19), F3-Knowledge about risk factors and causes (items 9 and 10), F4-Knowledge of self-treatment (items 11 and 12), F5-Knowledge about access to professional helpitems 13–15, and F6-Attitudes that facilitate seeking adequate psychological assistance, and attitudes toward psychological disorders (stigmatization; items 20–35). The scale score varies between 35 and 160.

### Statistical analysis

All data were statistically analyzed using SPSS Statistics for Windows (version 21.0; IBM, Armonkm NY). The descriptive analysis was conducted in all variables. The Pearson correlation test to confirm whether the PD and MHL were associated with MHSI. Then, a stepwise regression analysis was conducted to explore the factors associated with MHSI. Finally, Mediation analysis fully for all potential confounding factors were conducted. The independent variables were those with *p*-values < 0.05 in the demographic data. Further, in the Pearson correlation test, all independent variables that were significantly correlated with the dependent variable were input.

## Results

### Score of each item of help-seeking intentions

The scores for each item of MHSI are presented in Table 1. The total score (SD) of MHSI was 12.495(SD = 6.693; 95% CI, 31.738 ~ 34.211).

**Table 1:** Score of each item of the MHSI (*n* = 281, mean ± SD).

Item	Mean	SD	95% CI
Total	12.281	6.826	11.483 ~ 13.079
1. If I had a mental health concern, I would intend to seek help from a mental health professional.	2.580	0.903	2.474 ~ 2.686
2. If I had a mental health concern, I would try to seek help from a mental health professional.	2.858	0.891	2.753 ~ 2.962
3. If I had a mental health concern, I would plan to seek help from a mental health professional.	1.633	0.813	1.538 ~ 1.729

SD = standard deviation

### Demographic data and scores of MHSI

Demographic data on the participants MHSI are presented in Table 2. The analysis of the mean difference for MHSI showed a statistically significant difference in gender (*F* = 2.256, *p* = 0.002), age (*F* = 1.641, *p* = 0.004), marital status (*F* = 2.559, *p* = 0.000), degree level (*F* = 1.893, *p* = 0,013), economic status (*F* = 3.166. *p* = 0.000), and past psychological distress history by the question “Have you ever seen a mental health professional?” (*F* = 1.838, *p* = 0.017).

**Table 2:** Demographic data and scores of the help-seeking intentions (*n* = 281)

Variables	*N*(%)	Score (Mean ± SD)	*F*	*p*-value
Gender			2.256	0.002**
Male	175(62.28)	1.55 ± 0.50		
Female	106(37.72)	1.60 ± 0.55		
Age			1.641	0.044*
18–22	8(2.85)	2.67 ± 0.52		
23–28	128(45.55)	2.49 ± 0.58		
>28	145(51.60)	2.45 ± 0.50		
Marital Status			2.559	<0.001 *
Married	81(28.83)	2.80 ± 1.10		
Single	182(64.77)	3.57 ± 0.80		
Other	18(6.40)	3.20 ± 1.10		
Years in Singapore			1.362	0.141
< 0.5 year	52(18.51)	2.50 ± 1.60		
0.5-1 year	136(48.40)	4.09 ± 1.45		
1–2 years	75(26.69)	3.20 ± 1.37		
2–3 years	8(2.84)	3.37 ± 1.64		
3–4 years	4(1.42)	3.22 ± 1.66		
>4 years	6(2.14)	2.89 ± 1.83		
Degree level			1.893	0.013*
Undergraduates	15(5.33)	4.20 ± 1.34		
Master students	19(6.76)	4.60 ± 1.34		
Ph.D. students	158(56.23)	3.96 ± 1.38		
Post-doctors	20(7.12)	3.56 ± 1.10		
Visiting scholars	69(24.56)	3.50 ± 0.58		
Residence type			1.093	0.359
Off- campus accommodation	247(87.90)	1.15 ± 0.36		
On- campus accommodation	34(12.10)	1.12 ± 0.33		
Economic status			3.166	< 0.001 ***
Do not experience economic strain	204(72.60)	1.83 ± 0.99		
Experience economic strain	77(27.40)	2.27 ± 1.01		
Have you ever met a mental health professional?			1.838	0.017*
No;	262(93.23)	1.80 ± 0.45		
Yes, diagnoses but not treated;	9(3.20)	2.07 ± 0.26		
Yes, other treatment;	4(1.43)	2.73 ± 1.62		
Yes, treated with medication;	4(1.43)	2.15 ± 0.60		
Yes, treated with psychotherapy;	2(0.71)	2.75 ± 1.39		

SD = standard deviation

*p < 0.05;** *p* < 0.01; *** *p* < 0.001;

### Data of PD

Table 3 presents the mental health status. The total score of the PHQ-9 was 6.057 ± 5.469 (95% CI, 5.417 ~ 6.696) and the GAD-7 was 5.929 ± 4.729 (95% CI, 5.376 ~ 6.482). Out of the total participants, 11.74% exhibited moderate symptoms of depression, while 6.05% experienced moderate severe symptoms and 1.78% had severe depression. Additionally, 14.23% of the subjects demonstrated moderate symptoms of anxiety, and 6.05% displayed severe anxiety symptoms. The PHQ-9 and GAD-7 score showed significant differences on in gender (t_d_= 3.176, *p* < 0.001***; t_a_=2.110, *p* = 0.006**), age (F_d_=2.114, *p* = 0.005**; F_a_=2.517, *p* < 0.001***), marital status (F_d_=2.980, *p* < 0.001***; F_a_=4.015, *p* < 0.001**), years in Singapore (F_d_=2.603, *p* < 0.001**; F_a_=1.808, *p* = 0.025*), degree level (F_d_=2.289, *p* = 0.002**; F_a_=3.185, *p* < 0.001***), residence type (t_d_=4.435, *p* < 0.001***; t_a_=4.791, *p* < 0.001**), economic skills (F_d_=2.676, *p* < 0.001***; F_a_=3.320, *p* < 0.001***), and past psychological history by the question “Have been diagnosed or treated?” (F_d_=2.209,*p* = 0.003**; F_a_=3.689, *p* < 0.001***), respectively.

**Table 3:** Distribution of the PHQ-9 and GAD-7 categories

	Score range	*N*	%
PHQ-9			
Minimal depression	1 ~ 4	122	43.42
Mild depression	5 ~ 9	104	37.01
Moderate depression	10 ~ 14	33	11.74
Moderate severe depression	15 ~ 19	17	6.05
Severe depression	20 ~ 27	5	1.78
GAD-7			
Minimal anxiety	0 ~ 4	104	37.01
Mild anxiety	5 ~ 9	120	42.70
Moderate anxiety	10 ~ 14	40	14.23
Severe anxiety	> 15	17	6.05

t_d_ : *t* value of PHQ-9

t_a_ : *t* value of GAD-7

F_d_ : *F* value of PHQ-9

F_a_ : *F* value of GAD-7

### Correlation analysis of the Independent variables and MHSIS

Table 4 presents the correlation between the independent variables and the MHSIS. Significant positively correlations were observed between the PHQ-9 and GAD-7 scores and the MHSIS, with correlation coefficients of *r* = 0.272 and *r* = 0.220, respectively (*p* < 0.01). The mean total score of the MHLS was 98.644 ± 14.517 (95% CI: 96.947 ~ 100.342). Among the six dimensions of the MHLS, MHLS- attitude that promote recognition or appropriate help-seeking behavior (52.801 ± 9.665) and MHLS- knowledge of how to seek information (8.641 ± 4.419) showed relatively high scores. The MHSIS showed significant positive correlations with PHQ-9, GAD-7 and MHLS- A (*r* = 0.279, *r* = 0.200, *r* = 0.240, *p* < 0.01, respectively). Additionally, the MHSIS displayed a negative correlation with MHLS- H (r=-0.154, *p* < 0.01).

**Table 4:** Correlation analysis of the independent variables and MHSIS in international students in NUS (*N* = 281)

Independent variables	*r*	*p*
PHQ-9	0.279***	< 0.001
GAD-7	0.200**	0.001
MHLS- ability to recognize mental health disorders	-0.038	0.523
MHLS- knowledge of risk factors and causes	0.065	0.279
MHLS- knowledge of self-help treatment	-0.038	0.524
MHLS- knowledge of professional help available	-0.085	0.158
MHLS- knowledge of how to seek information	-0.154**	0.020
MHLS- attitudes promoting recognition and help-seeking behavior	0.240***	< 0.001
Total of the mental health literacy scale	0.084	0.161

*p < 0.05;** *p* < 0.01; *** *p* < 0.001

### Stepwise regression of factors influencing MHSI

A stepwise regression analysis was conducted with MHSIS scores as the dependent variable, and the results are presented in Table 5. Independent variables were *p*-values < 0.05, as determined by univariate analysis, and variable that showed a significant correlation with MHSIS in the correlation analysis. The variables that remained in the final model were PHQ-9 (β = 0.255; *p* < 0.001), MHLS- A (β = 0.182; *p* = 0.002), MHLS- H (β = -0.155; *p* = 0.006) and gender (β = -0.129; *p* = 0.021). The coefficient of determination R^2^ was 0.145, and these 4 factors accounted for 14.5% of the total variance of the MHSIS.

**Table 5:** Multiple linear stepwise regression of factors influencing the help-seeking intentions (*n* = 281)

Independent variable	B	se	β	*t*	*p*	*95%CI*
Constant	8.030	2.355	-	3.410	0.001**	-0.551 ~ 8.260
PHQ-9	0.318	0.072	0.255	4.415	< 0.001 ***	0.177 ~ 0.459
MHLS- A	0.128	0.041	0.182	3.152	0.002**	0.049 ~ 0.208
MHLS- H	-0.240	0.086	-0.155	-2.792	0.006**	-0.408~-0.071
Gender	-1.746	0.754	-0.129	-2.314	0.021*	-3.225~-0.267

* F* = 12.854, *P* = 0.000; *r* = 0.157, R^2^ = 0.145;

B = partial regression coefficient, se = standard error; β = standard regression coefficient

CI = confidence intervals

*p < 0.05; **p < 0.01; *** *p* < 0.001

### Mediation effect of MHL on the association between PD and MHSI

**Fig. 2:**
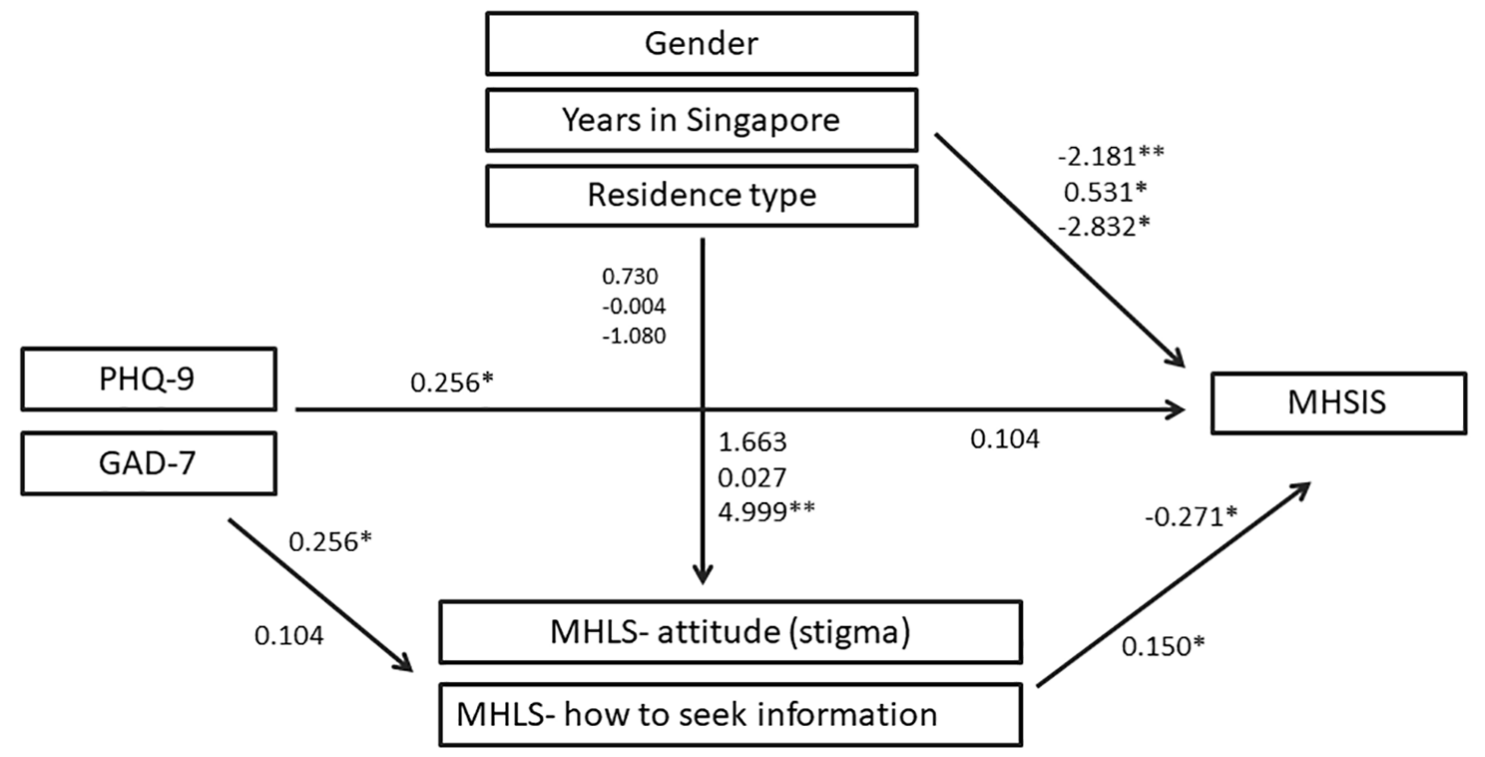
Hypothesis models of MHL and Demographic variables as a moderators of the relationship between psychological distress and mental help-seeking intentions

Results of the mediating effect between PD and mental MHSI are presented in Table 6. The total effects of the pathway from PHQ-9 = > MHLS- Stigma = > MHSIS, and from GAD-7 = > MHLS- H = > MHSIS were founded to be significant, supporting the presence and validity of the mediating role of MHL in the relationship between PD and mental MHSI. In this study, demographic variables were included as control variable in the mediating analysis. The results indicated the following mediating effects: gender (effect=-2.181**, *t*=-2.851), age (effect = 0.388, *t* = 0.529), marital status (effect=-0.290, *t*= -0.679), years in Singapore (effect = 0.531*, *t*=-2.152), degree level (effect=-0.502, *t*=-1.695), residence type (effect=-2.832*, *t*=-2.409), PHQ-9 (effect = 0.256*, *t* = 2.026), GAD-7 (effect = 0.104, *t* = 0.720), MHLS- H (effect=-0.271**, *t*= -3.125), MHLS- Stigma (effect = 0.150**, *t* = 3.576). Figure 2 illustrates the mediation analysis model for the association between the PD on MHSI.

**Table 6:** Mediating effects of MHL on the association between PD and MHSI

Effect	c	a*b	*SE*	95% CI		β	z	p	*c*
				Lower	Upper				
1	0.391**	0.038	0.024	-0.007	0.086	0.256*	1.610	0.107	0.256*
2	0.391**	0.097	0.044	0.008	0.179	0.104	2.195	0.028	0.256*
3	-0.013	-0.065	0.026	-0.067	0.003	-0.271**	-0.005	0.014	0.104
4	-0.013	-0.053	0.034	-0.063	0.008	-0.150**	-0.009	0.122	0.104

PHQ-9 = > MHLS- how to seek professional information = > MHSIS; insignificant mediation

PHQ-9 = > MHLS- attitude that promote recognition or appropriate help-seeking behavior (stigma) = > MHSIS; partial mediation

GAD-7 = > MHLS- how to seek professional information = > MHSIS; complete mediation

GAD-7 = > MHLS- attitude that promote recognition or appropriate help-seeking behavior (stigma) = > MHSIS; insignificant mediation

c: total effect

a*b: mediation effection

c: direct effect

## Discussion

In this study, we examined a mediation model involving PD, MHL, demographic variables, and MHSI among international students in NUS. The key findings of our research are as follows:

About one-fifth of the participants had above moderate depression symptoms and one-fifth had above moderate anxiety symptoms. Moderate levels of depression and anxiety rates are consistence across different gender, age, marital status, years in Singapore, Degree level, residence type and economic status sub-samples. The observed rates in our study are lower than those reported in a previous study, which indicated rates of 24.5% for depression and 20.7% for anxiety in Chinese international students studying in US colleges [39]. This disparity can be explained by several factors. Firstly, our sample consists of a higher proportion of Chinese international students, and their experiences in studying and living in Singapore may differ from those in other countries. Singapores cultural similarities to China in term of lifestyle, culture, diet, and customs may facilitate as smoother adaptation process, leading to a reduced likelihood of cultural shock and subsequently, less psychological stress [40]. But, our findings indicate that a considerable proportion of participants experienced depressive and anxiety symptoms. These results highlight the significance of addressing mental health concerns among international students in order to support their well-being and academic success.

Help-seeking intentions showed significant differences based on gender, age, marital status, degree level, economic status and past psychological distress history. Our research aligns with previous findings indicating that age and gender play a role in influencing intentions to seek professional psychological help [41]. Specially, women tend to have higher intention to seek help [42], possibly due to their more positive attitudes towards psychological openness. Additionally, our study revealed that younger individuals exhibited higher help-seeking intentions, and masters students displayed higher help-seeking intentions compared to doctoral students. Moreover, single individuals showed higher intentions to help-seeking than married individuals, and economic strain was found to increases help-seeking intentions. In our opinion, the social support and personality maturity of master students compare to doctors students are weaker, so they will seek more psychological assistance. Similarly, married individuals have more sources of social support than those without intimate relationships, such as interaction and support between partners and children, so the latter will seek more psychological assistance. These research findings are consistent with previous research results, which have also reported evidence for the association between age and help-seeking, with some studies showing negative associations [43, 44]. The previous study found that those with higher education were less likely to seek treatment [20]. Moreover, the prior studies showed that demographic variables, including economic and marital status, have been reported to influence peoples help-seeking for mental problems in various studies [45, 46].

Most of participants would definitely seek treatment if they had a future emotional problem, on the contrary, only one-fourth of students reported that they would seek treatment in the previous study [47]. Mental health literacy was significantly positively correlated with help seeking behaviour [48].Especially,female students with higher levels of depression and better knowledge of how to seek help are more likely to have stronger intentions to seek help, which the results was also founded in the previous study [49, 50]. Moreover, MHSI exhibited significant correlations with MHLS- A (stigma), aligning with results from prior studies [51]. A systemic review found evidence of the negative association between stigma and MHSI [52].These results suggest that individuals who recognize their mental health concerns are more likely to have stronger intentions to seek professional assistance. Conversely, those holding stigmatizing attitudes may display lower intentions to seek professional help. It is important to recognize that individuals with lower MHL may encounter barriers in recognizing and seeking appropriate help for their symptoms [53]. The inadequacy of an individuals MHL may lead to difficulty in identifying symptoms of mental illness, lack of understanding as well as negative attitude toward professional treatment, resulting in unfavorable attitudes toward seeking professional psychological help and lower adherence to treatment regimens [54]. consequently, international students with lower scores on these dimensions of MHL may experience higher levels of psychological distress and have lower intentions to seek help.

The results of the mediation analysis revealed significant total effects for the pathways from PHQ-9 = > MHLS- A = > MHSIS and from GAD-7 = > MHLS- H = > MHSIS, supporting the presence and validity of the mediating role of MHL in the relationship between PD and MHSI. Consistent with previous research, the results indicated a positive association between MHL and MHSI, MHL was significantly, positively correlated with general help-seeking intentions [56]. In the research by Clarie Goodfellow et al. [57], increased knowledge of treatment efficacy was associated with increased formal and informal help-seeking intentions whereas increased ability to identify specific mental health problems was associated with decreased formal and informal help-seeking intentions. Furthermore, the study revealed a negative association between MHL and stigma, which is consistent with existing literature [58]. Additionally, demographic variables were included as control variables in the mediating analysis, and gender, years in Singapore, and residence type exhibited both direct and indirect mediating effects in the model. These findings suggest that while depression directly affect MHSI, it also exerts a partial effect on MHSI by influencing MHLS- A. Similarly, anxiety directly influences MHSI, and it completely affects MHSI by influencing MHLS- H. As hypothesized, the results demonstrated the mediating roles of Depression, MHLS- A (stigma), and MHLS- H in the relationship between PD and MHSI, the results is contrary to the prior study [59]. Notably, the mediating effect of MHLS- H between PD and MHSI was found to be stronger than that of attitude (stigma), indicating its significant impact. Complete mediation results indicated that MHLS- H, which may be attributed to the notion that increased knowledge about how to seek professional assistance provides individuals with more options and choices for seeking help. On the other hand, partial mediation results indicated that MHLS- A enhanced help-seeking intentions, suggesting that addressing and improving attitude (reducing stigma) among international students can positively influence their willingness to seek help for mental health problems. These findings shed light on the pathway of influence between PD and MHSI, emphasizing the importance of improving attitude towards help-seeking among international students with depression and enhancing knowledge of how to seek professional information among students with anxiety. These insights provide valuable guidance for designing targeted interventions to promote MHSI among international students. Implementing measures to improve attitudes and enhance knowledge of professional resources may prove instrumental in empowering students to seek timely and appropriate mental health support, ultimately contributing to improved psychological well-being in this population.

In summary, this study explored the specific dimensions of MHL that influence the MHSI of international students in NUS experiencing PD. The findings indicated that the ability to seek professional service information and reduce stigma related to mental illness significantly impact their MHSI.

## Conclusion

The findings underscore the importance of promoting MHL and providing accessible resources and support for international students experiencing depressive and anxiety symptoms. Enhancing MHL can contribute to increased awareness, early recognition of symptoms, and effective help-seeking behaviors. Educational interventions and campus-wide initiatives should be implemented to address mental health concerns and improve the well-being of international students. Psycho-educational interventions targeted specifically for international students need to begin to address the barriers to accessing services, particularly with regards to the stigma of mental health difficulties. Interventions to increase help-seeking for mental problems by international students may be best served by focusing on special focus on males students, and those have economic strain, PD. Future research should focus on exploring the underlying factors contributing to the high prevalence of depressive and anxiety symptoms among international students and investigate effective strategies to improve mental health literacy and facilitate timely help-seeking.

## Strengths and limitations

This study makes a contribution by comprehensively examining the MHSI of international students at NUS from both psychological and cognitive perspectives (PD and MHL). However, several limitations should be acknowledged. Firstly, the cross-sectional design of the study prevents us from establishing causal relationship between the variables examined. Additionally, our analysis might not have considered all possible predictive factors necessitating future exploration.Such as the investigate the academic field that the participants study, the information would help to better understand the reason for seeking help or need for literacy. The limited scope of our sample, which only includes international students at NUS, warrants future research that incorporates a more diverse representation of international students.

## Data Availability

The datasets generated during and/or analyzed during the current study are available from the corresponding author on reasonable request.

## Abbreviations

NUS: National University of Singapore
MHSI: Mental Help-Seeking Intentions
MHL: Mental Health Literacy
MHLS: Mental Health Literacy Scale
MHLS- A: MHLS- attitude that promote recognition or appropriate help-seeking behavior (stigma)
MHLS- H: MHLS- knowledge of how to seek information
MHSIS: Mental Help Seeking Intention Scale
PHQ-9: Patient Health Questionnaire-9
GAD-7: Generalized Anxiety Disorder-7
